# Evaluation of the Clinical Course of Endogenous Endophthalmitis

**DOI:** 10.1177/24741264231191344

**Published:** 2023-08-18

**Authors:** Krista N. Thompson, Ahmed M. Alshaikhsalama, Angeline L. Wang

**Affiliations:** 1 The University of Texas Southwestern Medical Center, Dallas, TX, USA

**Keywords:** bacterial, endogenous endophthalmitis, endophthalmitis, fungal, infection, visual outcomes

## Abstract

**Purpose:** To evaluate the presentations, clinical course, treatments, and outcomes of endogenous endophthalmitis cases. **Methods:** Consecutive medical records from 2016 to 2021 of a county hospital and an academic, private hospital in Dallas, Texas were retrospectively reviewed. This study comprised 37 eyes of 31 patients with endogenous endophthalmitis. Collected data included demographic factors, identifiable risk factors, medical history, presenting symptoms, infectious data, complications, and best-corrected visual acuity (VA) throughout the clinical course. **Results:** Twenty-two eyes had bacterial endophthalmitis, 7 had fungal endophthalmitis, and 8 had infections that could not be classified. Of the bacterial cases, 5 eyes had panophthalmitis with associated cellulitis. The most common organisms were *Staphylococcus aureus*, *Candida albicans*, and *Pseudomonas aeruginosa*. The most common presenting symptoms were decreased vision (70%), eye redness (41%), and eye pain (38%). Among all cases, there was no significant difference in the presenting logMAR VA (1.86) before treatment and the most recent logMAR VA (1.75; *P* = .70) after treatment. However, fungal infections demonstrated better logMAR VA than bacterial infections 6 months after diagnosis (mean logMAR VA, 0.93 vs 2.54, respectively; *P* = .016) and at most recent follow-up (mean logMAR VA, 0.76 vs 2.3, respectively; *P* = .004). There was also a strong correlation between presenting VA and most recent VA (*r*^2^ = 0.81; *P* < .01). **Conclusions:** Visual outcomes of endogenous endophthalmitis cases were poor. Our study found 2 components to be predictive of final VA: (1) whether the infecting organism was bacterial or fungal and (2) a patient’s presenting VA.

## Introduction

Endogenous endophthalmitis is a rare but severe disease that arises when infectious agents spread hematogenously from extraocular sites into the eye. This infection can lead to irreversible vision loss and requires immediate medical attention due to its rapid progression and poor prognosis. Although only 5% to 15% of endophthalmitis cases are endogenous,^1^ those endogenous cases yield a worse visual prognosis than exogenous cases^2^ and thereby warrant specific study.

Common pathogens include gram-positive bacteria, gram-negative bacteria, and fungi,^3^ with bacterial cases being more common than fungal cases. Visual outcomes as an aggregate are poor.^4,5^ Previous studies have found that visual outcomes partially depend on the infecting organism.^1^ Most reports suggest that fungal infections yield a better prognosis for patients^6–8^; however, some studies have produced contradictory findings.^9,10^ Age, time to treatment, presenting visual acuity (VA), and pars plana vitrectomy (PPV) utilization have been implicated as prognostic factors by various studies.^3,7,8,11^ More research is warranted due to the limited number of patients and conflicting data.

Evaluation of the presentation, treatment, outcomes, and complications of endogenous endophthalmitis cases can provide diagnostic and prognostic information for this rare disease with a rapid and severe progression. This study aims to evaluate clinical presentations, clinical courses, treatments, and outcomes of endogenous endophthalmitis. The authors hypothesized that clinical outcomes would largely depend on the infecting organism.

## Methods

This study is a retrospective consecutive case series of a patient sample evaluated for endophthalmitis between January 1, 2016, and December 31, 2021, at a large public hospital and a large private hospital in Dallas, Texas. The review of medical records from the 2 hospitals resulted in 665 unique patients. All cases of endogenous endophthalmitis due to systemic infection with hematogenous ocular seeding were included, and cases of exogenous endophthalmitis due to ocular surgery or trauma as well as cases of other ocular pathologies were excluded. The sample comprised 37 eyes from 31 patients.

Data collected on patients with endogenous endophthalmitis included demographic factors (age, race, and sex), identifiable risk factors (immunosuppression status, chronic metabolic disease, diabetes, intravenous drug use, cancer, and surgery), medical history, presenting symptoms, infectious data (source of infection, method of identification, organisms, and antimicrobial susceptibility of organisms), best-corrected VA (BCVA) throughout the clinical course, enucleations, and complications (glaucoma, retinal detachments, and cataracts).

All laboratory testing and cultures, irrespective of source, were performed at the time of patient presentation to the emergency department or ophthalmology clinic. Data were collected from the laboratory results reported in the patient record.

Statistical tests assessed for significance in VA outcomes using *P* < .05 as a suitable significance value. BCVA was converted to logMAR for analysis. When comparing visual acuities of bacterial and fungal cases, only culture-positive cases were included in the calculations.

## Results

Between 2016 and 2021, 37 eyes of 31 patients were infected with endogenous endophthalmitis at the institutions studied. Seven eyes were infected with fungal endophthalmitis from 5 patients, and 22 eyes were infected with bacterial endophthalmitis from 18 patients. Of the bacterial cases, 5 eyes had panophthalmitis with cellulitis. Eight eye infections from 8 patients could not be definitively classified as bacterial or fungal. Demographic characteristics, disease laterality, and risk factors of the study population can be found in Table 1. The most common risk factor was diabetes.

**Table 1.** Demographic Characteristics of Study Patients.^a^

Characteristic	Bacterial Endophthalmitis (n = 18)	Fungal Endophthalmitis (n = 5)	All Patients (N = 31)^b^
Age and sex			
Age, y, mean (range)	59.6 (38-90)	58.6 (42-68)	57.5 (29-90)
Male, n (%)	7 (39)	3 (60)	15 (48)
Female, n (%)	11 (61)	2 (40)	16 (52)
Race and ethnicity, n (%)			
Black	2 (11)	1 (20)	4 (13)
White	15 (83)	4 (80)	23 (74)
Hispanic	6 (33)	3 (60)	13 (42)
Disease laterality			
Right eye	7	3	13
Left eye	7	0	13
Bilateral	4	2	6
Presence of a risk factor, n (%)^c^	17 (94)	5 (100)	30 (97)

a n and N = the number of patients.

b Eye infections from 8 patients could not be definitively classified as bacterial or fungal.

c Risk factors included immunosuppression, chronic metabolic disease, diabetes, intravenous drug use, history of malignancy, and history of invasive surgery.

**Figure 1.**
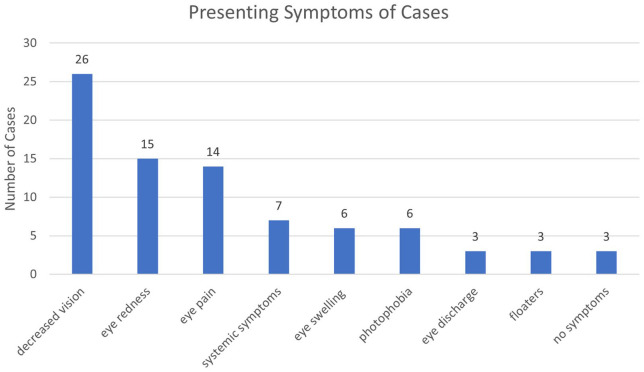
Presenting symptoms of patients with endophthalmitis. Number value indicates the number of cases. Systemic symptoms included nausea, vomiting, fever, unintentional weight loss, headache, chills, and body aches.

Presenting symptoms of the study population can be found in Figure 1. The most frequent presenting symptoms were decreased vision (70%), eye redness (41%), and eye pain (38%). All the patients who presented with eye discharge (n = 3) had confirmed bacterial endophthalmitis. Three eyes presented without any ocular or systemic symptoms. One of these patients had ocular symptoms in the other eye. Upon examination, the patient had bilateral endophthalmitis, so the infection was caught in the second eye prior to symptom onset. One patient had bacteremia, and there was a concern for ocular seeding, so an eye examination was performed prior to symptom onset. The last case had a waxing and waning mental status and thus was unable to report any symptoms if they were present. All patients with fungal endophthalmitis presented with symptoms.

Patients presented for medical care an average of 12 days (range, 0-60 days) after symptom onset. Ten patients presented for treatment within 24 hours (27%), and 4 patients presented for treatment more than 1 month after symptom onset (11%). Patients with bacterial endophthalmitis, on average, presented sooner after symptom onset (4.5 days) than patients with fungal endophthalmitis (32 days; *P* = .002).

**Figure 2.**
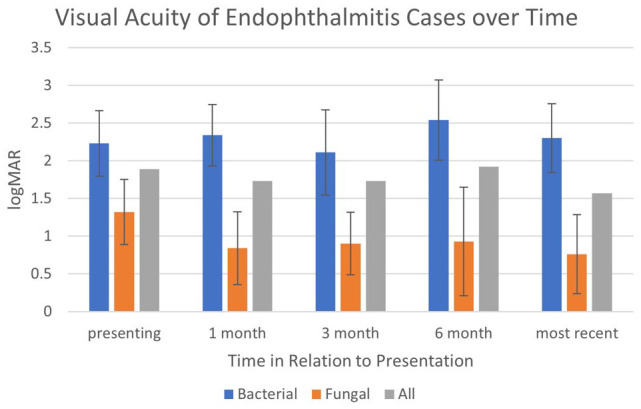
Visual acuity of endophthalmitis cases over time. Error bars represent the 95% confidence interval.

The mean logMAR VA at the time of presentation for all patients was 1.86 (range, 0.4-3). On average, patients with bacterial endophthalmitis presented with a logMAR VA of 2.2, while patients with fungal endophthalmitis presented with a logMAR VA of 1.3 (*P* = .024) (Figure 2).

Organisms were identified by blood or ocular culture for 30 of the 37 cases, yielding an identification rate of 81%. However, for one of these cases *Staphylococcus aureus* was found on blood culture, but the treating physician thought there was a possibility of fungal infection due to multiple white lesions on examination that could also be consistent with fungal endophthalmitis. This patient received treatment for both fungal and bacterial endophthalmitis; consequently, that case was not included in the calculations for fungal or bacterial endophthalmitis. The identification methods and identification rates can be found in Table 2.

**Table 2.** Utilization and Identification Rates of Various Cultures in 37 Cases of Endogenous Endophthalmitis.^a^

Identification Method Data	Blood Culture	Eye swab Culture	Vitreous Culture	Orbital Culture	Catheter-tip Culture	Aqueous Humor Culture
Times performed, No.	25	2	16	1	2	7
Causative organism isolated, No.	19	2	3	1	1	0
Causative organism identification rate, %	76	100	19	100	50	0

a These numbers correlate to the individual tests themselves, so patients with bilateral disease account for only 1 test performed.

There were a few instances in which organisms were identified from 2 different sources. Two cases identified the same organism from blood and vitreous fluid cultures. One case found methicillin-resistant *S aureus* (MRSA) on blood culture but *Enterobacter cloacae* on the catheter tip. MRSA was determined to be the causative agent. Another case found MRSA on blood culture but *Bacillus cereus* on vitreous culture. Despite the vitreous culture result, MRSA was determined to be the causative agent for this case, because the patient presented with MRSA bacteremia; *B cereus* was thought to be a contaminant. All other cases found only 1 organism via 1 method. Table 3 shows the causative organisms.

**Table 3.** Causative Organisms.

Organism	No. of Eyes (n = 29)
*Staphylococcus aureus*	8
*Candida albicans*	7
*Pseudomonas aeruginosa*	4
*Klebsiella pneumoniae*	3
*Roseomonas mucosa*	1
*Streptococcus pyogenes*	1
*Enterobacter cloacae* complex	1^a^
*Acinetobacter baumannii/nosocomialis* group	1^a^
*Escherichia coli*	1^a^
*Strep agalactiae*	1
*Serratia marcescens*	1
*Staph hominis*	1
*Staph epidermis*	1

a There was 1 instance of polymicrobial infection in which *Enterobacter cloacae complex*, *Acinetobacter baumannii*, and *Escherichia coli* were isolated from 1 case.

Susceptibilities were assessed for each of the identified organisms except for 1 patient with bilateral *Candida albicans* endophthalmitis. The susceptibilities for the most common infecting organisms are displayed in Tables 4 and 5.

**Table 4.** Susceptibilities of the Most Common Bacterial Organisms.^a^

Antimicrobial	*Staphylococcus aureus*		*Pseudomonas aeruginosa*		*Klebsiella pneumonia*	
	Tested, No.	Susceptible, %	Tested, No.	Susceptible, %	Tested, No.	Susceptible, %
Clindamycin	5	60	–	–	–	–
Oxacillin	5	60	–	–	–	–
Rifampin	5	100	–	–	–	–
Tetracycline	5	80	–	–	–	–
Trimethoprim + sulfamethoxazole	6	100	–	–	2	100
Vancomycin	7	100	–	–	–	–
Methicillin	7	43	–	–	–	–
Erythromycin	3	100	–	–	–	–
Daptomycin	4	100	–	–	–	–
Aztreonam	–	–	3	67	2	100
Cefepime	–	–	3	100	–	–
Ceftazidime	–	–	2	100	–	–
Ciprofloxacin	–	–	3	100	1	100
Gentamicin	6	100	2	67	2	100
Meropenem	–	–	3	100	–	–
Piperacillin/tazobactam	–	–	3	67	–	–
Tobramycin	–	–	3	100	2	100
Ampicillin + sulbactam	–	–	–	–	2	50
Ceftriaxone	–	–	–	–	2	100
Cefuroxime	–	–	–	–	2	100

a Cells with dashes indicate the antimicrobial was not analyzed. Bilateral disease from the same patient was counted only once. Cases were obtained from 2 facilities that tested different lineups of antimicrobials, accounting for the differences in the number tested values.

**Table 5.** Susceptibilities of *Candida albicans* Cases.^a^

Antimicrobial	*Candida albicans*	
	Tested, No.	Susceptible, %
5-Flucytosine	2	0
Amphotericin B	2	0
Anidulafungin	4	100
Caspofungin	4	100
Fluconazole	4	75
Itraconazole	2	50
Micafungin	4	100
Posaconazole	2	0
Voriconazole	3	67

a Bilateral disease from the same patient was only counted once. Cases were obtained from 2 facilities that tested different lineups of antimicrobials, accounting for the differences in the number tested values. One case of bilateral *Candida albicans* was not included because susceptibility testing was not performed.

The source of infection was often unknown. It was identified in only 16 of the 37 cases (43%). The most common sources of infection were catheter (n = 4 cases), urinary tract infection (n = 4 cases), and an infected central line (n = 3 cases). Other sources of infection included osteomyelitis, pyelonephritis, intravenous drug use, and liver abscess.

Treatment regimens for all cases of endogenous endophthalmitis can be found in Table 6. A total of 11 cases did not receive intravitreal antimicrobials. Patients who received intravitreal antimicrobials demonstrated better VA (mean logMAR VA, 1.44) at their most recent follow-up as compared with those who did not receive intravitreal antimicrobials (mean logMAR VA, 2.39), but this difference was not statistically significant (*P* = .09). The data were closer to reaching significance when removing fungal cases from the analysis (mean logmar VA, 1.63 with intravitreal antimicrobials vs 2.61 without intravitreal antimicrobials; *P* = .054).

**Table 6.** Treatment Regimens.^a^

Treatment	Bacterial	Fungal	All^b^
Antimicrobials			
Systemic	19	7	30
Intravitreal	14	6	27
Steroids			
Systemic	5	2	9
Topical	4	0	5
Unspecified	0	2	2
Procedures			
Pars plana vitrectomy	4	3	8
Enucleations	7	0	8

a The values indicate the number of eyes treated with the treatment regimen.

b Includes all eyes of patients with bacterial, fungal, and culture-negative endophthalmitis.

Of those who did not receive intravitreal antimicrobials, 4 eyes received enucleation and systemic agents. Two eyes were treated with enucleation and received no other treatment. Five eyes were not treated with enucleation. Of those five, 1 eye received systemic antibiotics and steroids, 1 received only systemic antibiotics, 1 received only steroids, and 2 received systemic antifungals.

Of those who underwent PPV, 1 patient with a bilateral PPV procedure was lost to follow-up. Another patient who underwent bilateral PPV died shortly after from another cause. Of the 4 other PPV procedures, 3 patients demonstrated no change in logMAR VA postoperatively. One patient’s logMAR VA improved by 0.9. There was no significant difference in the final logMAR VA of patients who underwent PPV compared with those who did not (mean logMAR VA, 1.82 vs 1.74, respectively; *P* = .89). Patients who received PPV were more likely to experience a retinal detachment (33% with PPV vs 3% without PPV; *P* = .048). None of the infections that resulted in enucleation were treated with PPV; however, there was no significant difference in enucleations compared with patients treated without PPV (0% with PPV vs 27.6% without PPV; *P* = .09).

The mean follow-up time was 11.3 months (range, 0-54 months). The overall mean logMAR VA 6 months after diagnosis was 2.11: in bacterial cases it was 2.54 while for fungal cases it was 0.93 (*P* = .016). The mean logMAR VA of the most recent visit was 1.75. Fifteen cases had a logMAR VA of 1.9 or worse at their most recent follow-up (39% of cases). Fourteen cases had a logMAR VA of 1.3 or better (37% of cases). The mean logMAR VA for bacterial cases at the most recent visit was 2.3, while the mean logMAR VA for fungal cases at the most recent visit was 0.76 (*P* = .004) (Figure 2). Among all cases, there was no significant difference in the presenting logMAR VA (1.86) before treatment and the most recent logMAR VA (1.75) after treatment (*P* = .70). Similarly, there was no significant difference in the presenting logMAR VA before treatment and the most recent logMAR after treatment for bacterial cases (2.23 vs 2.3, respectively; *P* = .84) and fungal cases (1.32 vs 0.78, respectively; *P* = .15).

A strong correlation existed between presenting and most recent VA (*r*^2^ = 0.81; *P* < .01). No correlation was found between the age of patients and most recent VA (*r*^2^ = 0.038; *P* = .30). Within bacterial cases, no correlation existed between time to presentation and most recent VA (*r*^2^ = 0.06; *P* = .46).

Of the most common organisms, mean final logMAR VA of *S aureus* infections was 2.14, of *C albicans* infections was 0.725, of *Pseudomonas aeruginosa* infections was 3, and of *Klebsiella pneumoniae* infections was 2.37.

There were a total of 8 enucleations performed for the 37 cases of endogenous endophthalmitis (22%). Seven of the enucleations were for confirmed bacterial cases (32% of the confirmed bacterial cases), and 1 case did not have an infectious organism identified. Three enucleations resulted from *S aureus* infections, 3 from *P aeruginosa* infections, and 1 from a *Serratia marcescens* infection. No enucleations were performed for confirmed fungal endophthalmitis cases. Five patients who underwent enucleation had orbital cellulitis in the infected eye. All of the enucleations were performed within 1 month of diagnosis.

There were postinfectious complications documented for 7 cases (19%). Three patients developed retinal detachments, 4 developed cataracts, and 2 developed glaucoma. Of those who developed retinal detachments, 2 had confirmed bacterial endophthalmitis. All of the patients who developed cataracts had confirmed bacterial endophthalmitis. One of the patients who developed glaucoma had fungal endophthalmitis while the other had bacterial endophthalmitis.

Eight patients, who accounted for 10 eyes in this study, died between infection and data collection. None of these deaths were attributed to endophthalmitis.

## Conclusions

In this study, we investigated the clinical characteristics and outcomes of patients presenting with endogenous endophthalmitis at a large public hospital and a large private hospital. As an aggregate, the patients in our sample did not demonstrate significant improvement in VA after treatment, indicating that the visual prognosis for endogenous endophthalmitis is poor overall. Our study found only 2 components to be predictive of final VA: (1) whether the infecting organism was bacterial or fungal, and (2) a patient’s presenting VA.

Patients presented with varied symptoms that prompted physician visitation or ophthalmologist evaluation. The most frequent presenting symptoms in our patient sample (ie, decreased vision, redness, and eye pain) were in concordance with previous studies (Figure 1).^12^ Approximately 97% of patients had at least 1 previously identified endophthalmitis risk factor, such as diabetes and malignancy (Table 1). Therefore, patients presenting with decreased vision, ocular pain, or redness and an associated risk factor should raise suspicion for potential endophthalmitis. Notably, 3 patients presented without any symptoms.

Two patients with fungal cases presented with systemic symptoms but without ocular symptoms. This finding demonstrates that patients with *Candida* fungemia may develop endophthalmitis without showing ocular symptoms. This information may suggest the necessity of screening those with *Candida* fungemia; however, the American Academy of Ophthalmology (AAO) recommends against regular screening for fungal endophthalmitis in patients with *Candida* fungemia due to overall low rates of fungemia resulting in endophthalmitis.^13^

We found that blood culture was the primary identification method (77% of those identified), with an overall identification rate of 81%. Some studies found similarly high positive culture rates for blood culture,^9,14^ but rates varied across studies.^4,15^ In our study, organisms were detected from vitreous culture at a rate of 19%, which was a substantially lower rate than that seen in previous studies.^7,16^

Cultures of the aqueous humor never yielded an organism within our study, indicating it may be an ineffective diagnostic method and may not benefit endogenous cases. Similarly, Feng et al^17^ found that organisms were more likely to be identified by vitreous culture than aqueous culture in endogenous and exogenous endophthalmitis cases. AlBloushi et al^18^ found that aqueous tap, although less sensitive and specific than vitreous tap, played an important role in identifying organisms when vitreous culture was negative for certain types of endophthalmitis. Within their study, however, aqueous humor culture was not efficacious for endogenous endophthalmitis and posttraumatic endophthalmitis.

The most prominent intravitreal agents used for bacterial and fungal endophthalmitis are vancomycin, ceftazidime, amphotericin B, and voriconazole. Among the patients with bacterial endophthalmitis, there was no resistance to vancomycin or ceftazidime. Among the patients with fungal endophthalmitis, 1 case of those tested (33%) was resistant to voriconazole, and 2 cases of those tested (100%) were resistant to amphotericin B. There have been case reports of endophthalmitis cases resistant to voriconazole.^19^ This resistance is concerning, as voriconazole is well tolerated, can treat a variety of fungal organisms, and is used when an organism shows high resistance to other antimicrobials.^20^ None of the patients in our sample were tested for resistance to both amphotericin B and voriconazole, so it is unclear whether any patients were resistant to both agents.

Our data suggest, in concordance with published results, that bacterial endophthalmitis has an acute onset and more severe presentation and prognosis than fungal-yeast endophthalmitis.^6–8^ Compared with patients with fungal endophthalmitis, BCVA was significantly worse in patients with bacterial endophthalmitis at all collected time points (*P* < .05), despite fungal endophthalmitis presenting much sooner after symptom onset. Therefore, patients with fungal endophthalmitis likely have a more favorable VA despite longer treatment delays. In our patient sample, 7 of 8 enucleations were from bacterial endophthalmitis, suggesting a worse prognosis with bacterial etiology.

Previous studies have indicated that the identified organism plays a role in the severity of endogenous endophthalmitis at presentation and its prognosis. Sridhar et al^21^ highlighted that within fungal species, infections with molds such as the *Aspergillus* species resulted in significantly worse VA than yeasts such as *C albicans*. Yet, patients with mold infections were also found to be in a worse state of health, which may complicate study findings. In our study, all identified fungal organisms were *C albicans*, which may have affected the prognosis of patients with fungal endophthalmitis. In contrast, the most common bacteria identified in our study (*S aureus*, *P aeruginosa*, and *Klebsiella* species) have all been shown to cause acute and severe ocular manifestations.^22,23^ Thus, it may be more representative to evaluate outcomes based on infectious organisms than taxonomic evolution.

Differences in BCVA could also have been exacerbated due to differences in treatment between fungal and bacterial endophthalmitis. In our study, patients with fungal endophthalmitis received intravitreal therapy at a rate of 83% vs the 64% of patients with bacterial endophthalmitis who received intravitreal antibiotics (Table 6). Although AAO recommends against ocular screening in patients with *Candida* fungemia in part due to no alternations in management,^13^ patients in this cohort with *Candida* endophthalmitis largely received intravitreal treatment.

The patients in our study who received intravitreal antimicrobials demonstrated better VA at the most recent follow-up than those who did not receive intravitreal antimicrobials regardless of the infecting organism, although this difference was not significant. Although improved outcomes among fungal cases cannot be solely attributed to intravitreal antimicrobials, this type of treatment may have been a factor. Intravitreal injection has not been systematically investigated but may be a factor in quickly subduing infection and improving visual outcomes. Therefore, the impact of intravitreal injection remains a worthy consideration in endophthalmitis treatment.

Multiple factors could prompt a physician to forego intravitreal treatment. If a patient presents with severe disease, the physician may send the patient for immediate enucleation because the eye is not salvageable. The use of intravitreal antimicrobials in such cases would be futile. Over half the patients who did not receive intravitreal antimicrobials received an enucleation. It was difficult to determine whether the physician decided against intravitreal treatment due to lack of viability. The visual difference between treatment groups may have been because a lack of intravitreal treatment was a proxy for necessary enucleation. With that in mind, the authors suggest a study focused on intravitreal treatment.

In concordance with other studies, we found presenting VA to be highly predictive of final VA, regardless of fungal or bacterial origin.^11^ This information is useful because VA is quick to obtain and can provide a rough prognosis prior to organism identification, which can take time to acquire. Because bacterial cases present with worse VA, it partially explains the visual outcome disparity between bacterial and fungal cases. Unlike other studies, the bacterial infections in our sample did not demonstrate better VA when treatment was administered sooner after symptom onset. The authors hypothesize that this was due to patients being infected with high virulence organisms, thus damage occurred rapidly. We also did not find age to be predictive of visual outcomes.^8^

Past studies have found that endophthalmitis cases treated with PPV demonstrated more useful visual improvement, better organism identification, and decreased levels of secondary intervention.^7,22,24,25^ In contrast, patients in our study who underwent PPV did not have significantly different final VA than those who did not undergo the procedure. Furthermore, other studies have found that PPV decreases the risk of developing a retinal detachment and requiring an enucleation/evisceration.^11,25^ Yet patients who underwent PPV in our sample were significantly more likely to develop a retinal detachment.

The prevalence of retinal detachments in patients treated with PPV could be due to significant vitritis or a consequence of the surgery itself. However, our study’s patients who required enucleation were not treated with PPV. There was no statistically significant difference in enucleations between the 2 groups, but this was likely due to sample size. Thus, PPV could have been useful for the patients in our sample by preventing enucleation, although it was not useful in improving visual outcomes or preventing retinal detachments. However, it is challenging to draw conclusions about PPV treatment efficacy in a study of this design due to physician bias in case selection.

There were limitations to our study. Our sample size was relatively small, with 37 eyes of 31 patients, which limits the ability to generalize the data. However, endogenous endophthalmitis is exceedingly rare, so similar studies have comparable sample sizes to ours.^8,11,26^ The small sample sizes underscore the need for multiple studies to enable broader generalizations from the data. Another limitation was the lack of access to information about patients’ baseline VA prior to infection.

Most patients did not receive ocular care at the 2 facilities in the study before their endophthalmitis infection, so they did not have prior visual acuities recorded on their medical records. The exclusion of this information limits our ability to quantify how much vision loss the patient experienced when they presented with their infection and how much vision was recovered from treatment. Patients in our study were also very sick with multiple co-morbidities, which could have impacted the patients’ visual outcomes and treatment options. Furthermore, the presentation, follow-up, and treatment of patients were quite variable. This variability limited how many patients could be included in different data analysis portions, particularly with patients lost to follow-up. Lastly, a causative organism was not identified for 8 cases, which limited some of the data analysis.

Future studies are warranted, particularly multi-institutional studies with increased sample sizes. There is also a need to study patients who had ocular care prior to infection with endogenous endophthalmitis to quantify the amount of vision lost when presenting with the infection. With the evident disparity between fungal and bacterial cases, future studies should work to characterize factors that predispose patients to fungal or bacterial endophthalmitis. The impact of intravitreal antimicrobial treatment also warrants further study.
